# Strike three, you’re out - when a primary psychosis just doesn’t cut it

**DOI:** 10.1192/j.eurpsy.2025.2037

**Published:** 2025-08-26

**Authors:** M. Vasconcelos, C. Silva, M. Bajouco, D. Mota, S. Batista, A. Mendes

**Affiliations:** 1 Psychiatry; 2 Neurology, Coimbra Hospital and University Centre, Coimbra, Portugal

## Abstract

**Introduction:**

Autoimmune psychosis has gained increased recognition as a distinct entity and is known to mimic variants of primary psychosis, typically presenting with an acute onset of polymorphic psychotic symptoms. We describe a case of probable autoimmune psychosis in a young patient who experienced a severe first psychotic episode.

**Objectives:**

Reflection over the diagnostic challenges of autoimmune psychosis.

**Methods:**

Clinical case report.

**Results:**

A 19-year-old male patient with no relevant medical history was admitted to the psychiatric ward due to a first psychotic episode with a peracute onset. The episode peaked with severe confusion, disorientation, and disorganized behavior, leading to his referral to the emergency room. This episode was characterised by delusional ideation with mystical, self-referential, and persecutory themes, complex auditory-verbal hallucinations, and marked negative and cognitive symptoms (including affective blunting, social withdrawal, apathy, alogia, impaired attention, decreased social cognition, and reduced speed of cognitive processing). The analytical study, substance screening, and brain CT upon admission were normal, leading to the assumption of a primary psychotic disorder. Antipsychotic therapy was initiated with progressive titration (risperidone, cariprazine, and clozapine), yet there was no significant improvement. Given the severe presentation and treatment resistance, a neurological examination was requested, which revealed no focal signs. A comprehensive laboratory workup showed positive ANAs, anti-recoverin antibodies, and hypocomplementaemia (C3 and C4). No significant abnormalities were observed in the brain MRI. CSF analysis revealed slight protein elevation (55 mg/dL) without pleocytosis, oligoclonal bands, or antibodies. EEG indicated mild to moderate encephalopathy with FIRDA bursts and focal paroxysmal activity in the left temporo-parieto-occipital region. Brain PET-FDG showed no significant abnormalities. Serum and CSF neurofilament levels were normal. Full-body CT and PET-FDG scans were also unremarkable. Given the findings, autoimmune psychosis was assumed. Treatment with IV immunoglobulin (30 g) and methylprednisolone (1 g for 5 days) was administered. The case was discussed in a multidisciplinary meeting, and a regimen of daily prednisolone (10 mg) was chosen. At follow-up, the patient showed slight improvement, with mitigation of the positive symptoms.

**Conclusions:**

Psychosis that does not respond to antipsychotic treatment and presents with atypical signs should raise suspicion of secondary immune-mediated schizophreniform psychosis. However, the challenge lies in identifying these patients, selecting appropriate diagnostic tests, and establishing criteria for implementing treatment.

**Disclosure of Interest:**

None Declared

